# Effects of Enriched Physical and Social Environments on Motor Performance, Associative Learning, and Hippocampal Neurogenesis in Mice

**DOI:** 10.1371/journal.pone.0011130

**Published:** 2010-06-15

**Authors:** Noelia Madroñal, Cristina López-Aracil, Alejandra Rangel, José A. del Río, José M. Delgado-García, Agnès Gruart

**Affiliations:** 1 Division of Neurosciences, Pablo de Olavide University, Seville, Spain; 2 Molecular and Cellular Neurobiotechnology, Catalonian Institute of Bioengineering and Department of Cell Biology, University of Barcelona and Centro de Investigación Biomédica en Red de Enfermedades Neurodegenerativas (CIBERNED), Barcelona, Spain; The Mental Health Research Institute of Victoria, Australia

## Abstract

We have studied the motor abilities and associative learning capabilities of adult mice placed in different enriched environments. Three-month-old animals were maintained for a month alone (AL), alone in a physically enriched environment (PHY), and, finally, in groups in the absence (SO) or presence (SOPHY) of an enriched environment. The animals' capabilities were subsequently checked in the rotarod test, and for classical and instrumental learning. The PHY and SOPHY groups presented better performances in the rotarod test and in the acquisition of the instrumental learning task. In contrast, no significant differences between groups were observed for classical eyeblink conditioning. The four groups presented similar increases in the strength of field EPSPs (fEPSPs) evoked at the hippocampal CA3-CA1 synapse across classical conditioning sessions, with no significant differences between groups. These trained animals were pulse-injected with bromodeoxyuridine (BrdU) to determine hippocampal neurogenesis. No significant differences were found in the number of NeuN/BrdU double-labeled neurons. We repeated the same BrdU study in one-month-old mice raised for an additional month in the above-mentioned four different environments. These animals were not submitted to rotarod or conditioned tests. Non-trained PHY and SOPHY groups presented more neurogenesis than the other two groups. Thus, neurogenesis seems to be related to physical enrichment at early ages, but not to learning acquisition in adult mice.

## Introduction

Interactions between an organism and its environment can lead to important neurobehavioral changes, and for several decades environmental enrichment (increasing sensory, motor, and cognitive stimulation) has been used to induce these changes in both intact and injured central nervous systems. The term “enriched environment” as an experimental process was introduced in the late 1940s by Donald Hebb [1]. Although there is no strict consensus on which environmental enrichment paradigms are the best [2], “enriched” animals are usually kept in larger groups and in big cages containing tunnels, nesting materials, toys, and running wheels that make the environment complex and variable. Molecular and cellular studies have demonstrated that these housing conditions result in both anatomical and physiological changes in the brain of animals subjected to them, compared with animals living in more-standard conditions. These changes include an increase in the total weight, the amount of protein content, and the thickness of the cerebral cortex [3], [4]. In this regard, the hippocampal region is one of the most interesting brain areas for determining the effects of enrichment on the neural tissue. Thus, it has been reported that enrichment increases hippocampal neurogenesis [5], the integration of these newly generated neurons into functional circuits [6], and the strength in the perforant path to the dentate gyrus [7] and in the CA3-CA1 [8] synapses.

The positive effects of environmental stimulation are not restricted to the cellular level, but also reach brain functioning. Behavioral studies have shown that exposure to enriched environments enhances memory capabilities in various tasks, particularly in spatial learning tests [5], [9], [10]. This enrichment-induced enhancement might be related to cellular effects on synaptic plasticity and hippocampal neurogenesis and survival. Firstly, it was shown that neurogenesis in dentate gyrus is important for certain forms of learning and memory [11], and this finding was strengthened when it was reported that these new neurons become synaptically integrated in hippocampal circuits [12] and mature into functional neurons [6].

Although it is assumed that environmental enrichment enhances learning and memory, this is a general assumption taken from the observation that these housing conditions also improve spatial memory performance. Nevertheless, little is known from a quantitative point of view regarding the effects of environmental stimulation on other associative learning tests in which the hippocampus is also involved. To determine whether an enriched environment has consequences on associative learning tests similar to those previously described for spatial learning, we studied here the effects of different living conditions on classical eyeblink conditioning as well as on instrumental conditioning, two associative learning paradigms in which the hippocampus seem to be involved [13]–[16]. Previous studies have been focused on the physical component of the enrichment, rather than on analyzing the putative social interactions when several animals are housed together. We included in our experimental design the study of the effects of social stimulation by itself, or in combination with physical enrichment. Furthermore, immunohistochemical studies were carried out in order to determine hippocampal cell proliferation and neurogenesis caused by the enriched environment. Our results indicate that not all types of learning are modified by a physically enriched environment, since motor abilities and instrumental learning are improved but the ability to acquire conditioned responses in a classical conditioning test is not modified. Our results also show that social stimulation is not a key factor for learning and memory improvement, although it is able to increase both hippocampal cell proliferation and neurogenesis, but only in very young animals.

## Results

### Environmental conditions prior to motor and associative learning tests

One week after arrival, 3-month-old animals were assigned randomly to one of the following four experimental groups: alone (AL), physical (PHY), social (SO), and social-physical (SOPHY). For the AL group, animals were placed in individual cages provided exclusively with the regular soft-wood mouse bedding. For the PHY group, animals were also placed in individual cages, but provided with a wheel, a tunnel, a ladder, a dummy mouse, and nesting material. For the SO group, animals were caged together (a total of 3 animals per cage), provided exclusively with mouse bedding. Finally, for the SOPHY group, animals were caged together (n = 3 per cage), but in an enriched environment, as for the PHY group. Animals were maintained in this situation for 30 days before the beginning of the experimental tests. For experiments, a total of 20 animals per group were selected at random for the rotarod test. Those animals were further divided for classical (n = 10 per group) and instrumental (n = 10 per group) conditioning. At the end of the behavioral study, animals were prepared for the immunohistochemical study (see Material and Methods).

In addition, 1-month-old animals were divided into the same four groups (n = 10 per group) and maintained for 30 days in the four types of environmental conditions. These animals were not used in any motor or learning test (AL-n, PHY-n, SO-n, and SOPHY-n). After the 30-day period, these animals were directly prepared for the immunohistochemical study, without any additional manipulation or training.

### Motor coordination following 30 days of training under the different environmental conditions

**Figure 1:**
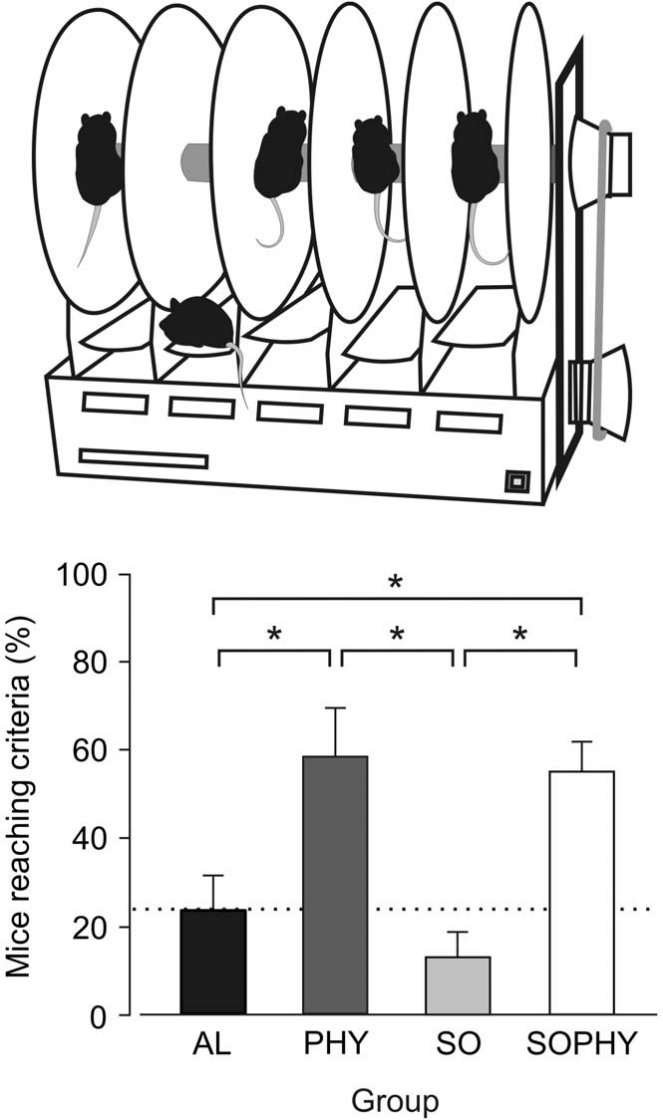
Percentage of animals from each experimental group that reached criterion on the accelerating rotarod. At the top is illustrated a diagram of the accelerating rotarod treadmill. The bars in the bottom diagram show the percentage (mean ± s.e.m.) of animals that reached criterion for the first experimental session. Note that performance for the two (physical, PHY, and social-physical, SOPHY) groups having an enriched environment presented significantly (*, *P*<0.001) larger values than for the other two (alone, AL, and social, SO) groups.

The rotarod test is a behavioral task assessing motor coordination performance. Fig. 1 illustrates the percentage of animals per group (AL, PHY, SO, SOPHY) that reached criterion during the first rotarod session. The selected criterion was to remain on the rod for ≥300 s without a fall. As shown, 60±11.3% (mean ± s.e.m.) of SOPHY and 56.7±6.6% of PHY mice reached the criterion during the first session. In contrast, only 24±8% of AL and 13.4±6.3% of SO mice reached the same criterion during the first session. The differences in rotarod performance during the first session were significantly different between the PHY/SOPHY groups and the AL/SO groups [*F*_(11,4)_ = 13.487; *P*<0.001; Fig. 1]. No significant differences were observed between the PHY and SOPHY groups (*P* = 0.678) or between the AL and SO groups (*P* = 0.246). By the 5th session, all PHY and SOPHY animals had already reached the selected criterion, whilst 42±12.5% of AL and 54±16.4% of SO mice reached criterion only during this session.

With regard to the mean time spent on the rod during a session per group, the PHY and SOPHY groups presented values significantly [*F*_(11,4)_ = 13.487; *P*<0.01] larger (261±45.3 s and 267±54 s) than those collected from the AL and SO (79±10.1 s and 114±25.9 s) during the first session. The differences were still significant (*P*<0.01) during the 5th session between the PHY (380±20.2 s) and the SOPHY (305±60.5 s) versus the AL (255±39.6 s) and the SO (270±46.5 s) groups.

In summary, animals with a physically enriched environment presented a better performance in the rotarod test than those placed in impoverished cages. The presence of social interactions has no positive effects on this task.

### Classical eyeblink conditioning in the four experimental groups

**Figure 2:**
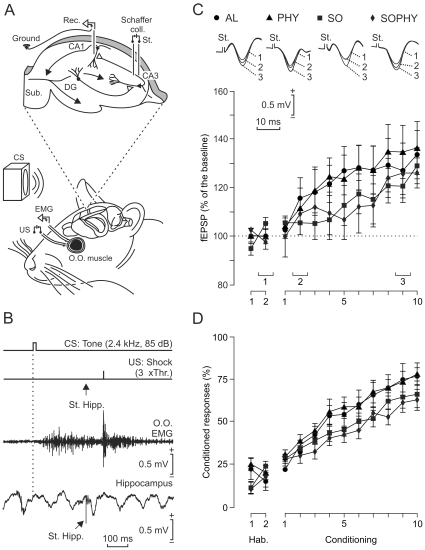
Quantitative analysis of the classical eyeblink conditioning test carried out with the four experimental groups. (A) Animals (n = 10 per group) were implanted with electromyographic (EMG) recording electrodes in the left orbicularis oculi (O.O.) muscle and with stimulating electrodes on the ipsilateral supraorbital nerve. Mice were also stimulated with tones delivered from a loudspeaker located 30 cm from the animal's head. As illustrated in the top diagram, animals were also implanted with stimulating (St.) and recording (Rec.) electrodes aimed to activate CA3-CA1 synapses of the contralateral hippocampus. (B) From top to bottom are illustrated the trace conditioning paradigm used in this study, and representative EMG and hippocampal records collected from the 10th conditioning session from a representative animal (AL group). For classical conditioning of eyelid responses, we used a tone (20 ms; 2.4 kHz; 85 dB) as conditioned stimulus (CS) and an electrical shock presented to the supraorbital nerve as unconditioned stimulus (US). The moment at which a single pulse (100 µs; square; biphasic) was presented to Schaffer collaterals is indicated by the arrow (St. Hipp.). Note the fEPSP evoked by the single pulse presented to Schaffer collaterals. (C) At the top are illustrated fEPSPs evoked at the CA3-CA1 synapse, 300 ms after CS presentation, in a selected animal from each group, during the 2nd habituation (1) and during the 2nd (2) and 9th (3) conditioning sessions. The graphs at the bottom show the evolution of fEPSP slopes (mean ± s.e.m.) during the successive sessions for alone (AL, black circles), physical (PHY, black triangles), social (SO, black squares), and social-physical (SOPHY, black diamond) groups. There were no significant differences between groups (*P* = 0.198). In contrast, fEPSPs evoked during conditioning were significantly larger from the 4th session on for the AL (*P*<0.001) and PHY (*P*<0.001) groups, and from the 7th to the 10th sessions for the SO (*P*<0.001) and SOPHY (*P*<0.002) groups, as compared with values collected during the habituation sessions. (D) The graphs illustrate the evolution of the percentage (mean % ± s.e.m.) of conditioned responses during the successive sessions for the four groups. Although there were no significant differences between groups (*P* = 0.078), the percentage of conditioned responses was significantly larger from the 2nd to the 10th conditioning sessions for AL (*P*<0.001) and PHY (*P*<0.002) groups, and from the 5th to the 10th sessions for SO (*P*<0.002) and SOPHY (*P*<0.002) groups, as compared with values collected during habituation sessions.

In a second series of experiments, we carried out the classical conditioning of eyelid responses in the four (AL, PHY, SO, and SOPHY) experimental groups (n = 10 animals/group) following the design illustrated in Fig. 2A. Figure 2B depicts some raw data collected from a representative AL animal. The mean percentage of conditioned responses (CRs) across the 10 days of trace conditioning for each experimental group is illustrated in Fig. 2D. In general, animals presented no evident changes in their performance during habituation sessions, whatever their experimental group. During conditioning sessions, mice displayed acquisition curves characterized by a sigmoid increase in the percentage of CRs ― from 29.5% (AL group) and 38% (PHY group) reached during the 2nd conditioning session to 62.5% (SOPHY group) and 78.4% (PHY group) during the 10th session. The percentage of CRs was significantly [*F*_(11,99)_ = 138.551; *P*≤0.002] larger from the 2nd to the 10th conditioning sessions for the AL and PHY groups, and from the 5th to the 10th sessions for the SO and SOPHY groups, as compared with values collected during habituation sessions.

Although the SO and SOPHY groups showed learning curves with lower values than those reached by the AL and PHY groups (Fig. 2D), no significant differences were observed across habituation (n = 2) and conditioning (n = 10) sessions in the percentage of CRs reached by the four experimental groups [*F*_(33,297)_ = 2.533; *P* = 0.78]. Thus, neither physical nor social enrichments had any beneficial effect on animal performance during the classical conditioning test.

### Evolution of field EPSPs across classical conditioning in the four experimental groups

Fig. 2C illustrates the slopes of fEPSPs evoked at the CA3-CA1 synapse across the successive habituation and conditioning sessions in the four groups of animals. Representative samples of fEPSPs collected from the four experimental groups across conditioning sessions are illustrated at the top of Fig. 2C. As already described in alert behaving mice [16], the slope of fEPSPs evoked at the CA3-CA1 synapse by single electrical stimuli presented to the Schaffer collaterals increased steadily across conditioning sessions in the four groups, reaching ≈120–130% of baseline values by the 8th-10th conditioning sessions. The slope (mV/s) of fEPSPs evoked across the successive conditioning sessions was significantly [*F*_(11,99)_ = 138.551; *P*≤0.002] larger from the 4th to the 10th sessions for the AL and PHY groups, and from the 7th to the 10th sessions for the SO and SOPHY groups, compared with values (considered as 100%) collected during the two habituation sessions.

In coincidence with what was described above for the percentage of CRs, the SO and SOPHY groups again yielded fEPSP curves with values slightly lower than those achieved by the AL and PHY groups. However, there were no significant differences between the four experimental groups [*F*_(33,297)_ = 1.219; *P* = 0.198] with respect to the slopes of fEPSPs evoked across conditioning sessions. According to these results, physical and social enrichments have no significant effects on the activity-dependent synaptic plasticity evoked at the CA3-CA1 synapse by classical eyeblink conditioning.

### Linear relationships between fEPSPs evoked at the CA3-CA1 synapse and the percentage of CRs reached across the successive conditioning sessions

**Figure 3:**
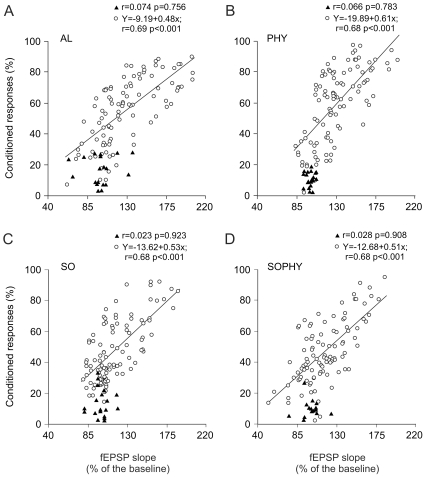
Quantitative analysis of the relationships between fEPSP slopes and the percentage of conditioned responses in the four experimental groups. (A)-(D). Data collected from animals (n = 10) included in the alone (AL, A), physical (PHY, B), social (SO, C), and social-physical (SOPHY, D) groups. Each point represents the mean value collected from a single animal during the corresponding session. Data illustrated were collected from habituation (black triangles) and conditioning (white circles) sessions. Regression lines and their corresponding equations are included exclusively for coefficients of correlation (r>0.6). The significance (*P*) values for each regression analysis are always indicated.

As illustrated in Fig. 3A-D, the slope of fEPSPs at the CA3-CA1 synapse evoked by stimulation of the Schaffer collaterals was linearly related (r≥0.68; *P*<0.001) to the percentage of conditioned responses across conditioning sessions, in the four experimental situations (slopes: AL, 0.48; PHY, 0.61; SO, 0.53; and SOPHY, 0.51), but not during habituation (r≤0.074; *P*≥0.756). The fact that the slopes of the four regression lines were similar suggests that the activity-dependent plasticity at the CA3-CA1 synapse was not affected by the physical and social enrichments to which the PHY, SO, and SOPHY groups were subjected, as compared with the AL group. As a whole, and confirming early findings [16], [17], this is an interesting finding, indicating the involvement of hippocampal intrinsic circuits in the step-by-step acquisition of an associative learning task.

### Operant conditioning abilities in the four experimental groups

**Figure 4:**
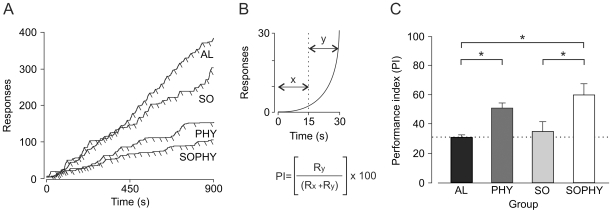
Quantitative analysis of the instrumental conditioning test (a fixed-interval schedule, 30 s) carried out with the four experimental groups. (A) Following a training period (3–7 days) of shaping to the Skinner box, using a fixed-ratio (1∶1) schedule, animals (n = 10 per group) were presented with the fixed-interval (FI30s) schedule in which they could obtain a food pellet by pressing the lever just after each 30-second period. The graph illustrates a cumulative record from a representative animal per group collected from the 9th training session. The x axis indicates session duration (s), whilst the y axis indicates lever presses (responses). The angled bars under the cumulative traces indicate reinforcements. (B) Animal performance during the FI30s schedule was determined with the illustrated performance index (PI) equation (Gottlieb et al. 2006). In this equation, Ry represents lever presses during the second part of the 30-second period and Rx represent presses during the first part (see diagram). The equation was designed to penalize lever presses during the first part, as well as>1 lever press during the second part. (C) Performance indexes (PIs) for the four experimental groups analyzed during the last (10th) conditioning session. Note that although alone (AL) and social (SO) groups presented higher rates of lever presses, they presented significantly (*P*≤0.001) lower PI values than groups with enriched environments, placed either alone (PHY) or with littermates (SOPHY) in the home cages.

Fig. 4A illustrates cumulative records collected from a representative animal of each experimental group during the 10th session of instrumental conditioning using a fixed-interval schedule (FR30s). Collected results indicate that animals of the different groups pressed the lever at different rates, suggesting that some of them were unable to learn an appropriate performance in response to this instrumental conditioning test. Specifically, SOPHY and PHY animals presented a lower mean number of lever presses than the other (AL and SO) two groups. In order to carry out a quantitative analysis of the animals' performance during this instrumental task, we used the analytical procedure illustrated in Fig. 4B (see ref. 15 for details). Indeed, the time (with respect to pellet delivery) at which the lever presses are concentrated is a good indication of the proper acquisition of this instrumental learning task. In order to establish the appropriate timing of lever presses in relation to the presentation of the reinforcement, we defined the performance index (PI) illustrated in Fig. 4B. This performance index favors lever presses concentrated in the second half (designated y, Fig. 4B) of the time period (30 s) assigned to pellet delivery. Optimum performance would be a single lever press carried out in the 15 s preceding pellet delivery. Upon applying the performance index to the collected data (Fig. 4C), it became evident that the best performance was achieved by the PHY (PI = 51.4±3.1) and SOPHY (PI = 58.2±7.6) groups, whose values during the 10th conditioning sessions were significantly different from those reached by the AL (PI = 33.9±1.3) and SO (PI = 34.2±6.3) groups [*F*_(6,54)_ = 5.420; *P*<0.001]. No significant differences were observed between the PHY and SOPHY groups (*P* = 0.182) or between the AL and SO groups (*P* = 0.517). In summary, physical, but not social, enrichment had a positive effect on animal capabilities of acquiring an instrumental task using a fixed-ratio schedule.

### Changes in hippocampal neurogenesis induced by the four experimental situations

To determine putative changes in neurogenesis in the dentate gyrus of rotarod-trained and instrumental-conditioned animals (AL, PHY, SO, and SOPHY groups), we developed an accumulative BrdU-pulse labeling (see Material and Methods for details). Interestingly, we were unable to observe any significant [*F*_(4,35)_ = 3.495; *P* = 0.016] difference between these four groups of animals 20 days after the BrdU labeling. Furthermore, the mean number of double-labeled NeuN-BrdU neurons was rather low: AL, 6.41±0.59; PHY, 5.53±0.88; SO, 3.35±0.85; and SOPHY, 5.12±0.82 (not illustrated). A possible explanation for this low number of double-labeled neurons, and for the lack of a significant effect of physical and/or social enrichment on dentate gyrus proliferation and survival of newborn neurons, is that these mice were too old at the time of the test (≈6 months at the time of the immunohistochemistry).

**Figure 5:**
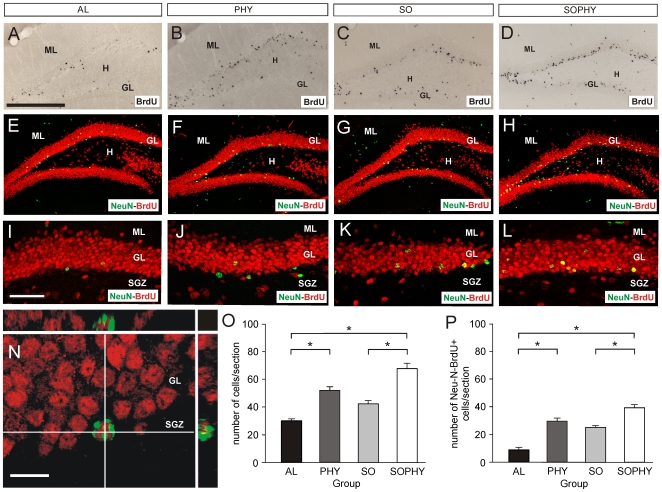
Analysis of cell proliferation and neurogenesis in the dentate gyrus of experimental groups. (A-L) Low-power photomicrographs illustrating BrdU-positive (A-D) and double-labeled BrdU/NeuN-positive cells (E-L) 20 days after the accumulative BrdU labeling in AL-n (A,E,I), PHY-n (B,F,J), SO-n (C,G,K), and SOPHY-n (D,H,L) groups. Notice the increased number of BrdU- and double-labeled cells in the SOPHY-n and PHY-n groups. A higher magnification of BrdU/NeuN-labeled cells in the suprapyramidal blade of the dentate gyrus of each experimental group is shown in I-L. (N) Example of a double-labeled cell in the subgranular zone (SGZ) of a mouse from the SOPHY-n group. The X and Y orthogonal projections of the photomicrograph are displayed (see Material and Methods for details). (O-P) Histograms showing the number (mean ± s.e.m.) of BrdU-positive cells (O) and double-labeled cells (BrdU-NeuN) in the SGZ + GL of the dentate gyrus in experimental groups. Asterisks indicate statistical differences between groups. (*P*≤0.001; ANOVA). Abbreviations: GL, granule cell layer; H, Hilus; ML, molecular layer. Scale bars: A = 200 µm pertains to B-H; I = 100 µm pertains to J-L, N = 10 µm.

To study this question further, we repeated the same BrdU test with young and non-conditioned experimental groups (AL-n, PHY-n, SO-n, and SOPHY-n). These animals were <3 months old at the time of immunohistochemistry. In this case, we obtained significant differences between groups. Firstly, no pycnotic nuclei were seen in BrdU-labeled cells in any of the experimental groups after the immunohistochemical processing. After DAB-Ni development, numerous BrdU-positive cells were observed in the subgranular zone in the PHY-n (Fig. 5B, O) and — especially — SOPHY-n (Fig. 5D, O) groups, whereas the AL-n (Fig. 5A, O) and SO-n (Fig. 5C, O) groups showed a low number of immunopositive cells. Similar observations were obtained after double immunostaining with NeuN and BrdU (NeuN-BrdU; Fig. 5E-N, P). The highest numbers of double-labeled NeuN-BrdU neurons were observed in the SOPHY-n (39.6±1.69) and PHY-n (29.66±2.04) groups. In contrast, the number of double-labeled neurons in the SO-n and AL-n groups was lower (25.4±1.45 and 9.1±0.59 respectively). Taken together, these data reinforce the notion that PHY-n, SO-n, and SOPHY-n experimental situations increased cell proliferation and neurogenesis compared with the AL group. In addition, mice caged together in an enriched environment (SOPHY-n) showed greater neurogenesis than those caged together without an enriched environment (SO-n) and mice caged alone under physical training (PHY-n). In conclusion, present data indicate an accumulative effect of the social and the physical training in an enriched environment with regard to cell proliferation and neurogenesis in the adult dentate gyrus of mice.

## Discussion

The present study was designed to determine the effects of an enriched environment on motor performance and on different forms of hippocampal-dependent learning. We also studied whether there is a causal relationship between these types of learning and hippocampal neurogenesis. The results show that motor performance and instrumental conditioning are improved by the physical factors of the enrichment, but the retention of classically conditioned memories is not. Furthermore, the improvement of some forms of learning does not seem directly related with the increase of newly generated neurons in the dentate gyrus of the hippocampus, since increasing social interaction is able to modify the number of hippocampal new neurons without affecting the learning paradigms assessed here.

Enriched environment usually includes increasing motor stimulation through access to running wheels. Several studies even indicate that this increased physical activity is a key factor of the enrichment, since the use of a wheel, without any other enriching element, is sufficient for regulating both learning and hippocampal neurogenesis [18]–[20]. In other cases, enrichment was achieved in absence of a running wheel [21]. However, those studies assessed spatial memory, without considering any other kind of learning, as, for example, associative learning tasks. Our results indicate that environmental enrichment, and presumably the use of the running wheel, can also affect motor coordination and improve performance in the rotarod test. Social interaction has no significant effect on the execution of this task.

Besides motor coordination performance, we also assessed two associative learning tests: trace classical eyeblink and instrumental conditionings. Although both paradigms are hippocampal-dependent, we found that physical enrichment has clear effects on operant conditioning abilities, without affecting the acquisition of classical conditioning of eyelid responses. Again, the social factor did not influence the learning processes. Taken together, spatial learning, rotarod, and instrumental conditioning are the learning paradigms in which acquisition is improved after exposure to enriched environments. According to these results, it can be hypothesized that only those forms of learning which require precise motor abilities are improved by an enriched environment. This is not the case of the classical conditioning of the eyelid responses, for which no special motor capacity is necessary.

The question of whether newly born neurons in the dentate gyrus of the adult hippocampus participate in learning and memory has been discussed for years [22]. Although is generally accepted that neurogenesis participates in hippocampal functions (e.g., [23], [24]), evidence for this connection still remains inconclusive (e.g., [25]). We firstly studied cell proliferation and neurogenesis in adult mice after rotarod and instrumental or classical conditioning, and we found no difference between groups living under the four different conditions selected. It has been reported that hippocampus-dependent learning affects adult-generated neurons — that is, there is an increase in the number of new cells in response to training in associative tasks that require the hippocampus, including a trace paradigm of the classical conditioning of the eyelid response [23], [24]. This would explain the fact that no difference in hippocampal neurogenesis was found between groups living under different environmental conditions after rotarod and classical or instrumental conditionings. In this regard, it has been shown that the survival and functional capabilities of adult-born dentate gyrus granule cells can be influenced by animal's training, when new-born granule cells are still in an immanture state [26], [27]. Finally, newborn neurons should be preferentially activated in hippocampal functions dependent on the dentate gyrus, such as pattern separation, as well as in discriminative learning tasks involving visual discrimination or spatial learning [28], [29]. In any case, our results cannot support the involvement of neurogenesis in the effects or enriched environment on a better motor performance or on the acquisition of selective associative learning tasks as instrumental learning.

However, differences in the number of adult-born dentate gyrus granule cells became evident when the immunohistochemical study was carried out in younger, non-conditioned mice. In fact, it is well known that neurogenesis declines with age [30]–[32]. Nevertheless, our results in this regard indicate that an increase in the number of new neurons does not always correlate with an improvement in learning capabilities, since neurogenesis was already activated by both physical and social enrichment, whilst only physically enriched environments were able to affect some learning performance tests. The way of establishing a causal relationship between new neurons and the acquisition of new memories could be by assessing learning after blocking neurogenesis. Various studies have attempted to deal with this question by reducing [33] or completely depleting [34], [35] the population of newly generated cells. Although those studies have reported deficits in various types of hippocampal-dependent learning task, including trace eyeblink conditioning [33], none of them has established beyond doubt that cognitive deficits are attributable exclusively to a loss of newly born cells in the dentate gyrus and not to effects on neural plasticity, to toxicity, or to inflammatory responses caused by the agents used for blocking neurogenesis [25]. In our experiments, animals housed in physically enriched environments presented more new hippocampal neurons than did those housed under social or standard conditions. However, this increased neurogenesis did not imply either a different way of acquiring a trace eyeblink paradigm, or a different activity-dependent synaptic plasticity evoked at the CA3-CA1 synapse by the learning process, suggesting that the previously described [33] effects of blocking hippocampal neurogenesis on this learning task is not due exclusively to the reduction in the number of adult-born cells. In this regard, it has also been reported that new hippocampal neurons are not required for spatial-learning improvement in animals living in an enriched environment [36].

Enrichment can also include increased social stimulation through larger numbers of animals per cage. Although it was claimed that social interaction alone cannot elicit the cerebral effects of enriched environments [37], that study did not cover neurogenesis. Since no recent study has focused on this issue, we included in our experiments the study of the effects of social enrichment — exclusively or in combination with physical stimulation — on both neurogenesis and learning. We found that social interaction increases hippocampal cell proliferation and neurogenesis, but is not able by itself to affect learning capabilities. Definitively, our results provide more evidence that neurogenesis is not the way by which environmental enrichment alters hippocampal-dependent behavior.

## Materials and Methods

### Subjects

Experiments were carried out on male C57Bl/6 mice obtained from an official supplier (Granada University Animal House, Granada, Spain). Animals were 1 or 3 months old at the moment of arrival, weighing 17±3 and 25±2 g respectively. Before experimental manipulations, animals were housed in separate cages (n = 10 per cage). All mice were kept on a 12/12 h light/dark cycle with constant ambient temperature (21±1°C) and humidity (50±7%). Food and water were available *ad libitum*. Electrophysiological and behavioral studies were carried out in accordance with the guidelines of the European Union Council (2003/65/European Union) and current Spanish regulations (Boletín Oficial del Estado 252/34367-91, 2005) for the use of laboratory animals in chronic experiments. Experiments were also approved by the ethical committee of the local institution (Universidad Pablo de Olavide, Comité Ético de Experimentación Animal, code 07/4/20/12/2007).

### Rotarod

In this study, we used an accelerating rotarod treadmill for mice (Ugo Basile, Varese, Italy; Fig. 1). Animals (n = 20 per group) were placed on the rod and tested at 36 rpm for a maximum of 400 s on each of 5 consecutive days. Between trials, mice were allowed to recover in their home cages. The total time that each mouse was able to stay on the rod was recorded automatically by a trip switch under the floor of each rotating drum, and computed as the latency to fall. Results were averaged to obtain a single value for each group and session [38]. The percentage of animals per group able to stay on the rod for ≥300 s without a fall was computed as a criterion.

### Classical eyeblink conditioning

Mice assigned for classical conditioning of eyelid responses (n = 10 per group) were prepared as follows. Firstly, they were anesthetized with 0.8–1.5% isoflurane, supplied from a calibrated Fluotec 5 (Fluotec-Ohmeda, Tewksbury, MA) vaporizer, at a flow rate of 1–4 L/min oxygen (AstraZeneca, Madrid, Spain) and delivered by a mouse anesthesia mask (David Kopf Instruments, Tujunga, CA). Once anesthetized, animals were implanted with bipolar recording electrodes in the left orbicularis oculi muscle and with stimulating electrodes on the ipsilateral supraorbital nerve. Electrodes were made of 50 µm, Teflon-coated, annealed stainless steel wire (A-M Systems, Carlsborg, WA, USA). Electrode tips were bared of the isolating cover for 0.5 mm and bent as a hook to allow a stable insertion in the upper eyelid. In the same surgical step, mice were also implanted with bipolar stimulating electrodes in the contralateral (right) Schaffer collateral/commissural pathway of the dorsal hippocampus (2 mm lateral and 1.5 mm posterior to bregma, and 1–1.5 mm from the brain surface [39]) and with a recording electrode placed in the right CA1 stratum radiatum (1.2 mm lateral and 2.2 mm posterior to bregma, and 1–1.5 mm from the brain surface). These hippocampal electrodes were made of 50 µm, Teflon-coated tungsten wire (Advent Research, Eynsham, UK). The final location of the hippocampal recording electrode was determined according to the field potential depth profile evoked by single pulses presented to the Schaffer collateral pathway [16]. A bare silver wire affixed to the skull served as a ground. All the implanted wires were soldered to two four-pin sockets (RS Amidata, Madrid, Spain). Bone screws and dental cement fixed the cannula and the sockets to the skull (see Fig. 2A).

Experimental sessions were carried out with three animals at a time. Animals were placed in separate small (5×5×10 cm) plastic chambers located inside a larger (30×30×20 cm) Faraday box. Both the electromyographic (EMG) activity of the orbicularis oculi muscle and fEPSPs were recorded with Grass P511 differential amplifiers (Grass-Telefactor, West Warwick, RI, USA), at a bandwidth of 0.1 Hz-10 kHz. A high-impedance probe (2×10^12^ Ω, 10 pF) was used for fEPSP recordings.

Animals were classically conditioned using a trace paradigm. For this, a tone (20 ms, 2.4 kHz, 85 dB) was presented as a conditioned stimulus (CS), whilst the unconditioned stimulus (US) consisted of a 500 µs, 3× threshold, square, cathodal pulse applied to the supraorbital nerve 500 ms after the end of the CS (Fig. 2B). A total of two habituation and 10 conditioning sessions (one session per day) were carried out for each animal. A conditioning session consisted of 60 CS-US presentations, and lasted ≈30 min. For a proper analysis of CRs, the CS was presented alone in 10% of the cases. CS-US presentations were separated at random by 30±5 s. For habituation sessions, only the CS was presented, also for 60 times per session, at intervals of 30±5 s. As criterion, we considered a “conditioned response” the presence, during the CS-US interval, of EMG activity lasting >20 ms and initiated >50 ms after CS onset. In addition, the integrated EMG activity recorded during the CS-US interval had to be at least 2.5 times greater than the averaged activity recorded immediately before CS presentation [40].

During habituation and conditioning sessions, fEPSPs were evoked in the CA1 area by single 100 µs, square, biphasic (negative-positive) pulses applied to Schaffer collaterals 300 ms after CS presentation (Fig. 2B, C). Pulse intensity was set at 35–45% of the amount necessary to evoke a maximum fEPSP response (range, 0.05–0.15 mA) — that is, well below the threshold for evoking a population spike [16], [34]. An additional criterion for selecting stimulus intensity was that a second stimulus, presented 40 ms later, evoked a larger (>20%) synaptic field potential than the first [35], [41]–[43].

At the end of the experiments, and in order to determine the location of hippocampal electrodes, mice included in this test were deeply re-anesthetized (4% chloral hydrate solution, 10 mL/kg) and perfused transcardially with saline and 4% phosphate-buffered paraformaldehyde. Brains were dissected out, postfixed overnight at 4°C, and cryoprotected in 30% sucrose in PBS. Brain sections were obtained in a microtome (Leica, Wetzlar, Germany) at 50 µm. Selected sections including the dorsal hippocampus were mounted on gelatinized glass slides and stained using the Nissl technique with 0.1% toluidine blue.

### Instrumental conditioning

A total of 10 animals per group were assigned for operant conditioning. Training and testing took place in basic Skinner box modules (n = 3) measuring 12.5×13.5×18.5 cm (MED Associates, St. Albans, VT, USA). The operant chambers were housed within a sound-attenuating chamber (90×55×60 cm), which was constantly illuminated (19 W lamp) and exposed to a 45 dB white noise (Cibertec, S.A., Madrid, Spain). Each Skinner box was equipped with a food dispenser from which pellets (Noyes formula P; 45 mg; Sandown Scientific, Hampton, UK) could be delivered by pressing a lever. Before training, mice were handled daily for 7 days and food-deprived to 80% of their free-feeding weight. Shaping took place for 15 min during successive days, in which mice were shaped to press the lever to receive pellets from the food tray using a fixed-ratio (1∶1) schedule. Animals were maintained on this 1∶1 schedule until they reached the selected criterion — namely, until they were able to obtain ≥20 pellets for 2 successive sessions. Mice reached criterion after 4–7 days of training (see ref. 15 for details).

Once criterion for the 1∶1 schedule was reached, conditioning was carried out for 10 days using a fixed-interval (FI30s) schedule. For this, the first lever press carried out by the mouse after each period of 30 s was rewarded with a pellet. Each session lasted for 15 min. The start and end of each session was indicated by a tone (2 kHz, 200 ms, 70 dB) provided by a loudspeaker located in the recording chamber. Conditioning programs, lever presses, and delivered reinforcements were controlled and recorded by a computer, using a MED-PC program (MED Associates, St. Albans, VT, USA). Those animals were further used for the immunohistochemical study (see below).

### Immunohistochemical study

To determine cell proliferation and neurogenesis in the hippocampus of trained mice, a daily BrdU (50 mg/kg b.w., Sigma-Aldrich) pulse by intraperitoneal injection was administered for four days (Fontana *et al.* 2006). In a first set of experiments we injected animals (n = 10 per group, AL, PHY, SO, SOPHY) that received instrumental conditioning. In this case, BrdU injections were carried out during the four days following the end of the experiments. These animals were maintained for 20 additional days in the same physical and environmental situation comprising the survival period after proliferation when the newborn cells develop differentiated phenotypes [44]. In a second set of experiments, non-conditioned animals (AL-n, PHY-n, SO-n, SOPHY-n) received four BrdU injections (i.p.) and were allowed 20 additional days in their home cages before the immunochemical study.

BrdU-injected mice were sacrificed 20 days after the last BrdU injection. Animals were perfused with 2% buffered paraformaldehyde, and fixed brains were post-fixed in the same fixative solution for an additional 2.5 hours. After fixation, brains were cryoprotected in 30% sucrose dissolved in 0.1 M PBS, and frozen in isopentane solution for storage at −80°C. Brains were coronally sectioned (30 µm thick), using a freezing microtome (Leica), and were further processed for immunocytochemistry. For BrdU immunohistochemistry, sections were processed essentially as described [45]–[47]. Briefly, sections were pre-treated for 15 min with cold 0.1 N HCl and 2 N HCl at 37°C for 20 min to denature DNA. After rinsing in 0.1 M PBS, they were incubated with a Fab goat anti-mouse IgG (1/50 diluted; Jackson Immunocytochemical) for 2 h and incubated with primary anti-BrdU antibodies (Rat monoclonal antibody anti-BrdU (diluted 1∶50, Harlan Sera-Lab, Loughborough, England). Tissue-bound primary antibody was detected using a biotinylated secondary antibody and the ABC method with DAB-Ni as chromogen [48]. Parallel sections were stained with cresyl violet or processed for the double immunofluorescence detection of BrdU and NeuN (diluted 1∶50; Chemicon) with Alexa-Fluor 488 and Alexa-Fluor 568 tagged secondary antibodies (Molecular Probes, Eugene, USA). Sections were mounted on Fluoromount and analyzed on an Olympus Fluoview SV 500 confocal microscope. All images were obtained in sequential scanning laser mode to avoid fluorochrome cross-excitation, and further processed with the Silicon Graphics Imaris software to obtain three-dimensional orthogonal projections. For the sake of homogeneity, a total of 20 sections per brain were analyzed. Data were quantified as mean number of neurons/section±s.e.m.

### Data collection and analysis

In the case of classical conditioning experiments, EMG and hippocampal activities and one-volt rectangular pulses corresponding to CS and US presentations were stored digitally on a computer through an analog/digital converter (CED 1401 Plus; Cambridge Electronic Design, Cambridge, UK), at a sampling frequency of 11–22 kHz and an amplitude resolution of 12 bits. Commercial computer programs (Spike 2 and SIGAVG, from Cambridge Electronic Design) were modified to represent EMG and fEPSP recordings. Data were analyzed off-line for quantification of CRs and fEPSP slopes with the help of homemade representation programs [16], [40], [49], [50]. Field EPSP slopes were collected as the first derivative (V/s) of fEPSP recordings (V). For this, five successive evoked field synaptic potentials were averaged, and the mean value of the slope was determined for the rise time period (i.e., the period of the slope between the initial 10% and the final 10% of the evoked field potential).

For the rotarod test, collected data (latencies to fall, session, and animal) were collected by computer and stored for off-line analysis [38]. For operant conditioning, cumulative records of lever pressings and pellet rewards were stored on-line on a computer connected to the Skinner boxes.

Computed results were processed for statistical analysis using the Sigma Stat for Windows package (SPSS 13.0 for Windows package (SPSS, Chicago, IL, USA). Unless otherwise indicated, data are represented by the mean ± s.e.m. Collected data were analyzed using a two-way ANOVA test, with time or session as repeated measure, coupled with contrast analysis when appropriate. One-way ANOVA allowed checking the statistical differences between different groups. Regression analysis was used to study the relationship between the fEPSP slope and the percentage of CRs.
